# The complete chloroplast genome of *Cyrtosia nana* (Rolfe ex Downie) Garay and its phylogenetic analysis

**DOI:** 10.1080/23802359.2025.2528572

**Published:** 2025-07-09

**Authors:** Ding-Xiang Yu, Hai He

**Affiliations:** a Chongqing Academy of Agricultural Sciences, Chongqing, China; b College of Life Sciences, Chongqing Normal University, Chongqing, China

**Keywords:** Cyrtosia nana, mycotrophic, chloroplast genome, phylogeny

## Abstract

*Cyrtosia nana* is a mycotrophic orchid distributed in southwestern China and Thailand. Here, we sequenced and assembled its complete chloroplast (cp) genome using next-generation sequencing (NGS) and established a robust phylogenetic framework, providing a foundation for further studies on evolution of related taxa. The complete chloroplast genome was 85,766 bp, including a pair of inverted repeat regions (12,999 bp), a small single-copy region (14,744 bp), and a large single-copy region (45,024 bp). It encodes 47 unique genes, including 28 protein-coding genes, 16 tRNA genes, and 3 rRNA genes. Phylogenetic analysis revealed that *C. nana* was closely related to *C. lindleyana* and *C. septentrionalis*.

## Introduction

*Cyrtosia nana* (Rolfe ex Downie) Garay 1986, is a beautiful mycotrophic orchid distributed in southwestern China and Thailand (Garay 1986; Chen et al. 2009). It had fleshy or sometimes tuberlike roots, reddish brown stems, pale yellow flowers with a bright yellow lip (Chen et al. 2009). According to the study by Barrett et al. (2014), the plastids of specialized heterotrophic plants still retain the chloroplast genome, but with varying degrees of gene loss. With rapid development of the second-generation sequencing technology, the chloroplast genome information was widely used for studying taxonomy, phylogeny, evolution, conservation and ecology in plants (Zhang et al. 2022). Until now, only two plastid genomes of *Cyrtosia* have been reported (Kim et al. 2019; Zhou et al. 2023), which has greatly hindered the aforementioned studies in this genus. This study used whole-genome Illumina sequencing technology to obtain the full-length chloroplast genome sequence of *C. nana*, and compared it with the chloroplast genome of several other species in the subfamily Vanilloideae, to reveal the evolutionary relationship of *C. nana* and its position in the phylogeny of *Cyrtosia*.

## Materials and methods

### Materials

**Figure 1.**
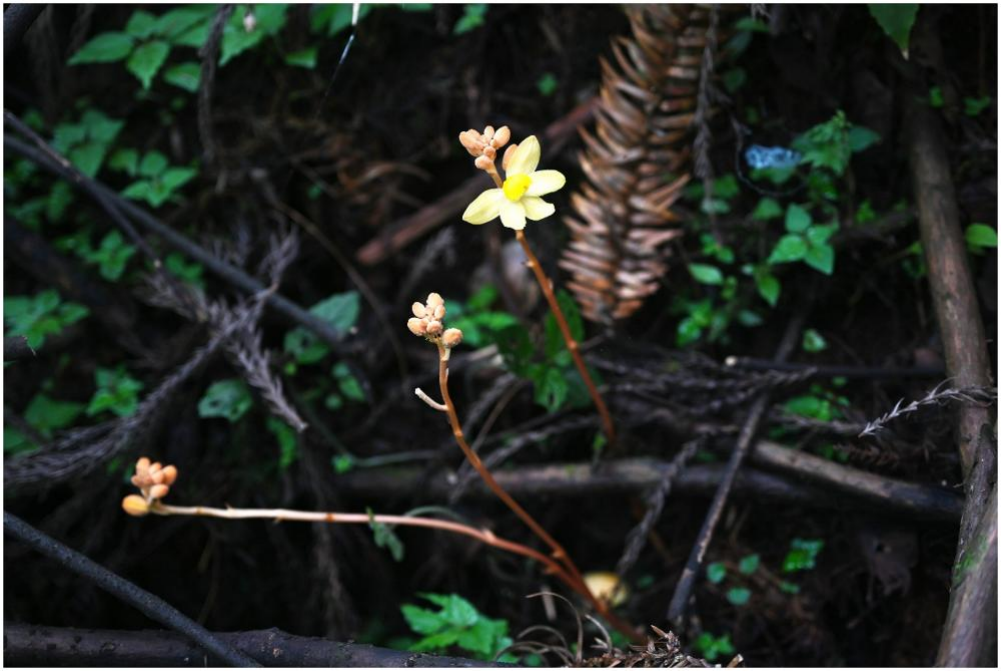
Field picture of *Cyrtosia nana*, the photo taken by Ding-Xiang Yu. *C. nana* grows under the moist forest of China cedar (*Cryptomeria japonica* var. *sinensis*). Plants small, ± fleshy. Rhizome short, stout. Stem erect, yellowish white, slightly tinged with red, 10–22 cm tall. Flowers pale yellow, lip with orange-red longitudinal stripes.

The samples of *Cyrtosia nana* (Figure 1) were collected from Chongqing, China (JIangjin District: 28.6007° N, 106.4464° E) and temporarily preserved in silica gel. Voucher specimens were deposited in the Herbarium of Chongqing Nature Museum (CQNM) under voucher specimen number *ZY500116101* (contact person: Ding-Xiang Yu, Email: yutengshu@163.com).

### DNA extraction, amplification and sequencing

Extracting DNA from silica-gel dried leaves using (Doyle and Doyle 1987) protocol. Paired-end sequencing libraries were meticulously constructed with insert sizes of approximately 350 bp, and the sequencing was conducted at the Beijing Genomics Institution (BGI; Shenzhen, China).

### Chloroplast genome assembly, annotation

∼10Gb of raw data were filtered by fastp v0.23.2 (Chen et al. 2018) with default parameters and assembled using GetOrganelle v1.7.6.1 (Jin et al. 2020). Chloroplast genome was annotated and manual corrections by Geneious Prime v2023.1.2 (Biomatters Ltd., Auckland, New Zealand) and Geseq (https://chlorobox.mpimp-golm.mpg.de/ geseq.html) based on the cp genome of *Cyrtosia septentrionalis* 1986 (Rchb. F.) Garay (MH615835). Chloroplast genome map generated using CPGview (http://www.1kmpg.cn/cpgview). The annotated chloroplast genome was submitted to the GenBank under the accession number PV083754.

### Phylogenetic analysis

The phylogenetic tree was constructed based on 28 protein-coding genes (PCGs) shared by 12 cp genomes of the Vanilloideae, and *Apostasia wallichii* R. Br. 1830 (Apostasioideae), as outgroup species. The sequences alignments were generated by MAFFT v7.508 (Katoh and Standley 2019). Filtering of ambiguously aligned and low mutation sites using Gblocks v0.9b (Talavera and Castresana 2007) with default parameters. The nucleotide substitution model for the matric was estimated using the software jModelTest v.2.1.6 (Posada 2008) and the best fit model (GTR+F + R3) was selected using the corrected Akaike information criterion (AIC). A maximum-likelihood (ML) method for phylogenetic analysis was performed *via* IQ-Tree v1.6.10 (Nguyen et al. 2015) and visualized in FigTree v1.4.4 (http://tree.bio.ed.ac.uk/software/figtree).

## Result

**Figure 2.**
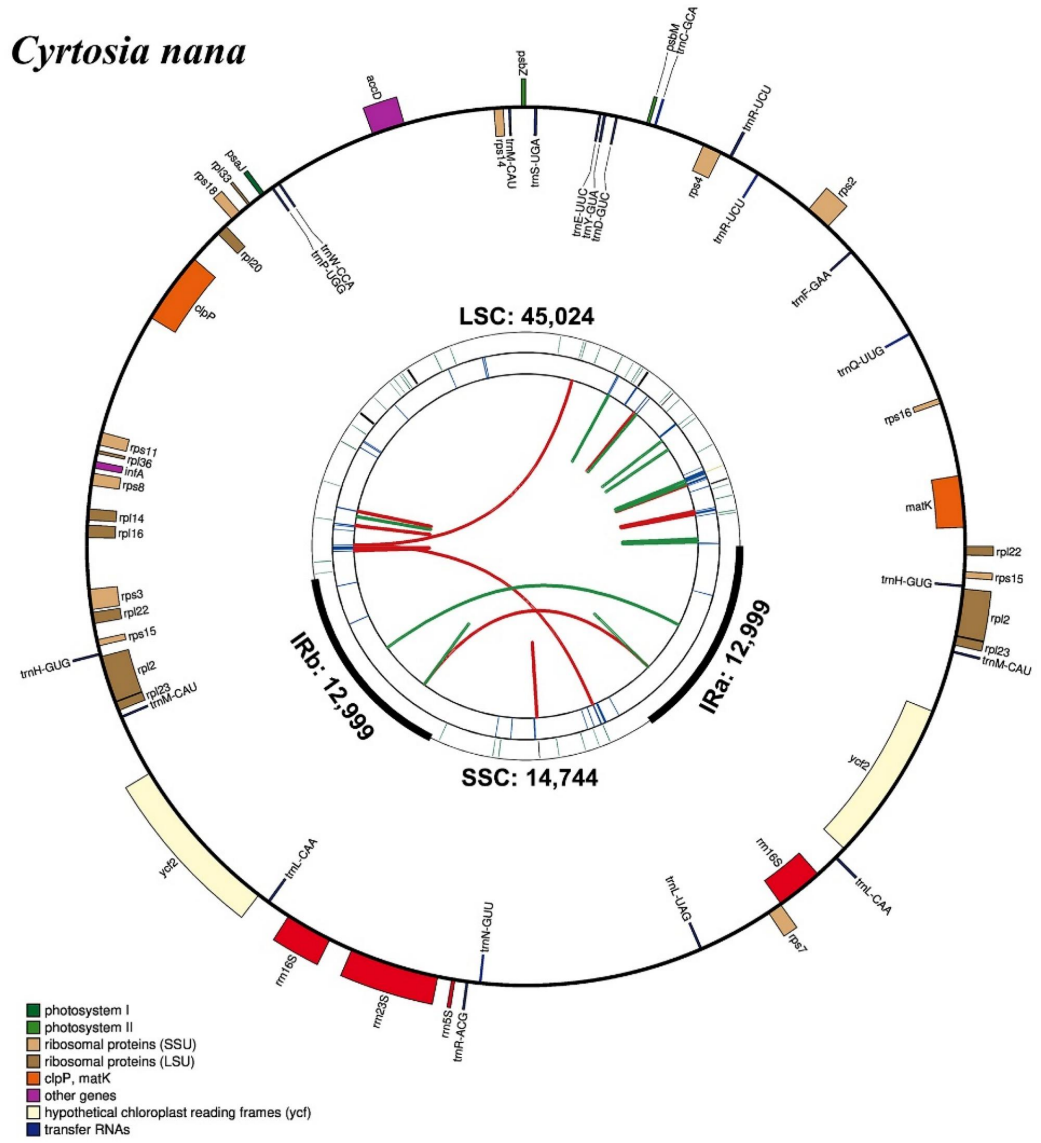
Chloroplast genome map of *Cyrtosia nana*. The map was generated by CPGView. Genes with different functions are shown in different colors. Genes within circles are transcribed clockwise and genes outside circles are transcribed counterclockwise. LSC: large single-copy region; SSC: small single-copy region; IR: inverted repeat.

**Figure 3.**
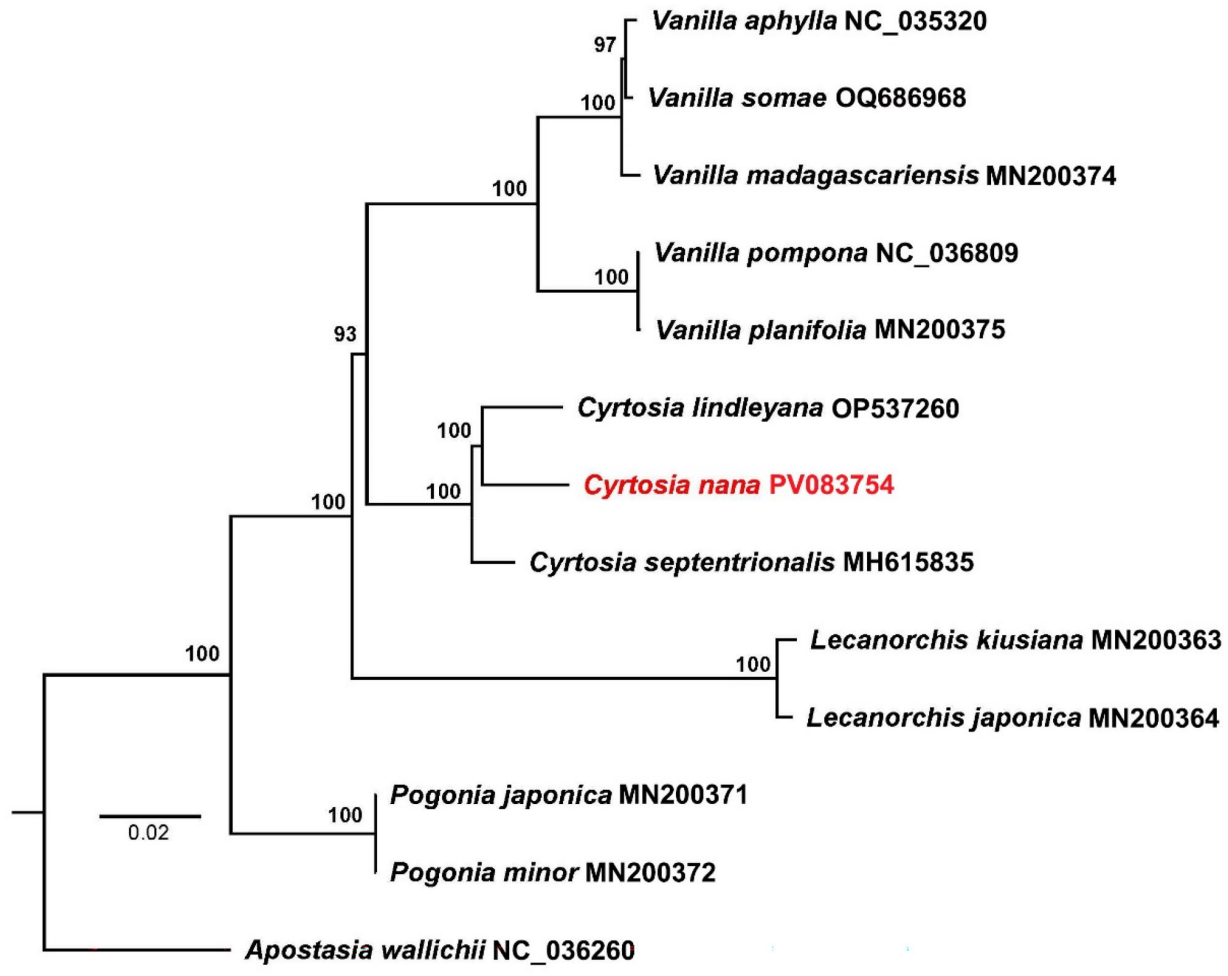
The ML phylogenetic tree for *Cyrtosia nana* based on 13 cp genomes in Orchidaceae. The accession numbers of used sequences follow the species names, and the newly sequenced genome is shown in red font. Sequences used for tree construction were as follows: *Apostasia wallichii*, NC_036260 (Niu, Pan, et al. 2017); *Pogonia minor*, MN200372 (Kim et al. 2020); *P. japonica*, MN200371 (Kim et al. 2020); *Lecanorchis japonica*, MN200364 (Kim et al. 2020); *L. kiusiana*, MN200363 (Kim et al. 2020); *Cyrtosia septentrionalis*, MH615835 (Kim et al. 2019); *C. lindleyana*, OP537260 (Zhou et al. 2023); *Vanilla planifolia*, MN200375 (Kim et al. 2020); *V. madagascariensis*, MN200374 (Kim et al. 2020); *V. pompona*, NC_036809 (Amiryousefi et al. 2017); *V. aphylla*, NC_035320 (Niu, Xue, et al. 2017); *V. somae*, OQ686968 (Divakaran and Rafieah 2021).

The raw data are available in the National Center for Biotechnology Information (NCBI, https://www.ncbi.nlm.nih.gov/) under the BioProject PRJNA1213241. The average coverage for the assembled cp genome was 817× (Figure S1). The complete chloroplast genome of *Cyrtosia nana* is 85,766 bp in length, containing a quadripartite structure that consists of a large single-copy (LSC) region of 45,024 bp and a small single-copy (SSC) region of 14,744 bp with two inverted repeat (IR) regions of 14,744 bp (Figure 2). The overall GC content was 34.8%, which is higher than either LSC region (30.7%), but lower than the SSC (37.4%) or IR (40.6%) regions. A total of 59 genes were annotated (47 unique), including 4 rRNA genes (3 unique), 21 tRNA genes (16 unique), and 34 protein-coding genes (28 unique). Introns were detected in 3 genes, where 2 genes (*rpl2* and *ycf1*) had a single intron, and the *clpP* genes had two introns (Figure S2). The phylogeny reconstructed based on 28 PCGs shared by 12 species in the subfamily Vanilloideae strongly supports the fact that 3 species of *Cyrtosia* formed a monophyletic group, and *C. nana* is closely clustered with *C. lindleyana* Hook. f. & J. W. Thomson in Hooker 1855 (Figure 3).

## Discussion and conclusions

In this study, the complete chloroplast genome size of *Cyrtosia nana* was only 85,766 bp, it was much smaller than the average of sequenced land plants (151 kb; Zhang et al. 2022). However, similar to the two published plastid genomes in this genus, their chloroplast genomes underwent a large degree of gene loss (Kim et al. 2019; Zhou et al. 2023). For example, *NDH* genes (except the truncated gene *ndhB* in *Cyrtosia septentrionalis*) were lost (Table S1), which was reported as the result of their evolution to adapt to the extreme light-free environment of the understory in the with conduplicate-leaved genera (*Phragmipedium*, *Mexipedium* and *Paphiopedilum*) (Liao et al. 2024). In addition, compared to other two species, genes of *atpA*, *atpB*, *atpE*, *petG*, *petL*, *psaA*, *psbC*, *rbcL*, *rpl32*, *rpoA*, *rpoC2*, *rps12* were also loss in *C. nana*. The inferred phylogenetic tree analysis also confirmed for the first time the phylogenetic position of *C. nana* as a member of the genus *Cyrtosia* in the subtrib. Vanillinae of the subfamily Vanilloideae of Orchidaceae. Within the genus, *C. nana* and *C. lindleyana* were sister to each other with high supported values, and then grouped with *C. septentrionalis.* This first reported cp genome of *C. nana* is a significant addition to the plastid genomes for the whole *Cyrtosia* genus, elucidates the evolutionary position of *C. nana* and lays the groundwork for subsequent studies on the phylogeny. Further studies will reveal the relationship between the genera *Cyrtosia* and *Galeola,* and *Cyrtosia lindleyana* was documented in the Chinese flora as *Galeola lindleyana* (Hook. f. & J.W. Thomson) Rchb. f. (Chen et al. 2009).

## Supplementary Material

Supplemental Material

## Data Availability

The data that support the findings of this study are openly available in GenBank number PV083754 (https://www.ncbi.nlm.nih.gov/nuccore/PV083754) and the related BioProject, raw sequencing files in SRA, and the Bio-Sample number are PRJNA1213241, SRR32064690 and SAMN46321526 (https://www.ncbi.nlm.nih.gov/), respectively.
