# Supplementary material for: The complete chloroplast genome of Cyrtosia nana (Rolfe ex Downie) Garay and its phylogenetic analysis
Source: Mitochondrial DNA B Resour. 2025 Jul 9;10(8):683–6. doi: 10.1080/23802359.2025.2528572 (PMC12243006; doi:10.1080/23802359.2025.2528572)
Supplement: Supplemental Material [file TMDN_A_2528572_SM7785.docx]

**Figure S1.** The read coverage depth map of the assembled genome.


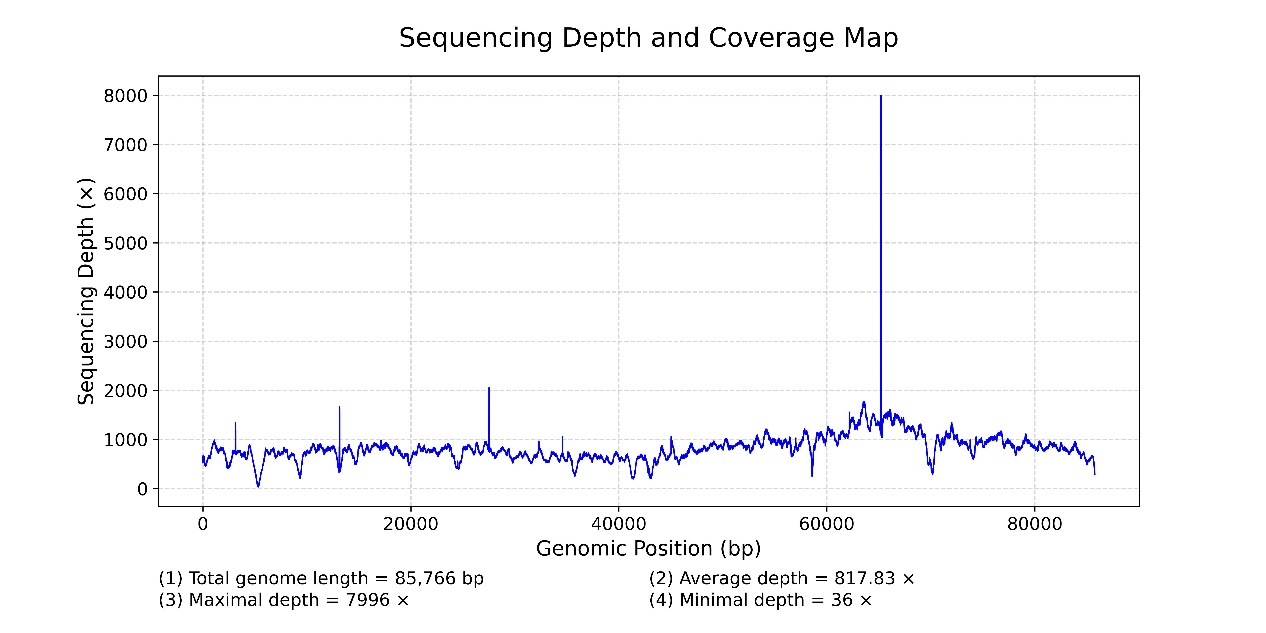


**Figure S2.** Schematic maps of cis-splicing genes in the chloroplast genome of *L. paradoxum*. Maps generated using CPGView. The gene names are shown on the left, and the gene structures are on the right.


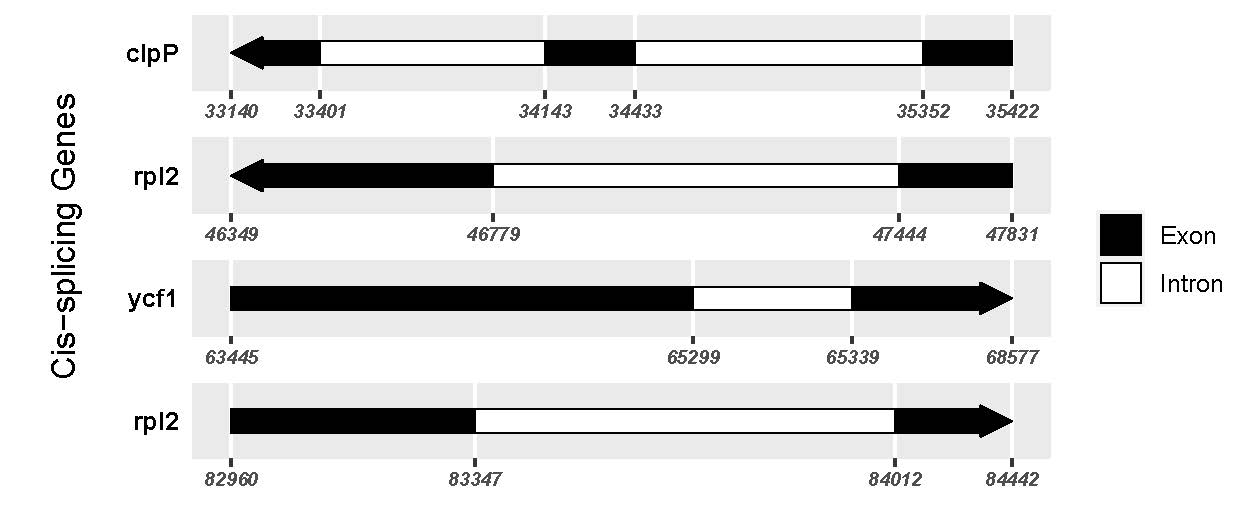


**Table S1.** A comparative analysis of gene loss in three *Cyrtosia* species.

| *Cyrtosia nana* | *Cyrtosia lindieyana* | *Cyrtosia septentrionalis* |
| --- | --- | --- |
| accD | accD | accD |
|  | atpA | atpA |
|  | atpB | atpB |
|  | atpE | atpE |
|  |  | atpF |
|  |  | atpH |
|  |  | atpI |
| clpP | clpP | clpP |
| infA | infA | infA |
| matK | matK | matK |
|  |  | ndhB |
|  | petD |  |
|  | petG | petG |
|  | petL | petL |
|  | psaA | psaA |
|  | psaB |  |
|  | psaI |  |
| psaJ | psaJ | psaJ |
|  | psbA |  |
|  |  | psbB |
|  | psbC | psbC |
|  | psbD |  |
| psbM |  | psbM |
|  | psbK |  |
| psbZ | psbZ | psbZ |
|  | rbcL | rbcL |
| rpl14 | rpl14 | rpl14 |
| rpl16 | rpl16 | rpl16 |
| rpl2 | rpl2 | rpl2 |
| rpl20 | rpl20 | rpl20 |
| rpl22 | rpl22 | rpl22 |
| rpl23 | rpl23 | rpl23 |
|  | rpl32 | rpl32 |
| rpl33 | rpl33 | rpl33 |
| rpl36 | rpl36 | rpl36 |
|  | rpoA | rpoA |
|  | rpoC2 | rpoC2 |
| rps11 | rps11 | rps11 |
|  | rps12 | rps12 |
| rps14 | rps14 | rps14 |
| rps15 |  | rps15 |
| rps16 | rps16 | rps16 |
| rps18 | rps18 | rps18 |
| rps19 | rps19 | rps19 |
| rps2 | rps2 | rps2 |
| rps3 |  | rps3 |
| rps4 | rps4 | rps4 |
| rps7 |  | rps7 |
| rps8 | rps8 | rps8 |
| ycf1 |  | ycf1 |
| ycf2 | ycf2 | ycf2 |
|  | ycf4 |  |
| rrn16S | rrn16 | rrn16 |
| rrn23S | rrn23 | rrn23 |
|  | rrn4.5 | rrn4.5 |
| rrn5S | rrn5 | rrn5 |
| trnC-GCA | trnC-GCA | trnC-GCA |
| trnD-GUC | trnD-GUC | trnD-GUC |
| trnE-UUC | trnE-UUC | trnE-UUC |
| trnF-GAA | trnF-GAA | trnF-GAA |
|  | trnfM-CAU | trnfM-CAU |
|  |  | trnG-GCC |
|  | trnG-UCC | trnG-UCC |
| trnH-GUG | trnH-GUG | trnH-GUG |
|  | trnI-CAU | trnI-CAU |
|  |  | trnI-GAU |
|  | trnK-UUU | trnK-UUU |
| trnL-CAA | trnL-CAA | trnL-CAA |
| trnL-UAG | trnL-UAG | trnL-UAG |
|  |  | trnL-UAA |
| trnM-CAU | | trnM-CAU |
| trnN-GUU | trnN-GUU | trnN-GUU |
| trnP-UGG | trnP-UGG | trnP-UGG |
| trnQ-UUG | trnQ-UUG | trnQ-UUG |
| trnR-ACG | trnR-ACG | trnR-ACG |
| trnR-UCU | trnR-UCU | trnR-UCU |
|  |  | trnS-GCU |
|  |  | trnS-GGA |
| trnS-UGA | trnS-UGA | trnS-UGA |
|  |  | trnT-GGU |
|  | trnT-UGU | trnT-UGU |
| trnW-CCA | trnW-CCA | trnW-CCA |
| trnY-GUA | trnY-GUA | trnY-GUA |
